# COVID-19: Facts, Cultural Considerations, and Risk of Stigmatization

**DOI:** 10.1177/1043659620917724

**Published:** 2020-04-21

**Authors:** Debra Pettit Bruns, Nina Vanessa Kraguljac, Thomas R. Bruns

**Affiliations:** 1 The University of Alabama, Tuscaloosa, AL, USA; 2 The University of Alabama at Birmingham, AL, USA; 3 CW4 (R) US Army, Birmingham, AL, USA

**Keywords:** coronaviruses, COVID, respiratory

## Abstract

Data on COVID-19 supports targeted social distancing could be an effective way to reduce morbidity and mortality, but could inadvertently increase stigma for affected populations. As health care providers we must be aware of the facts of COVID-19, cultural implications, and potential for stigmatization of populations affected by COVID-2019. It is important to consider the real economic impact related to lost workdays due to quarantine and social isolation efforts as well as travel restrictions that may negatively impact access to care and ability to pay for care. Efforts geared towards general education about the disease and the rationale for quarantine and public health information provided to the general public can reduce stigmatization.

Countries who are successful at aggressive screening, early identification, patient isolation, contact tracing, quarantine, and infection control methods should also address the risk of stigmatization among populations and the negative effects which could occur. The cases of COVID-19 will continue to rise and the virus will be sustainable for future infections. Timely and appropriate public health interventions addressing cultural impact and risk for stigmatization along with proper screening, treatment, and follow up for affected individuals and close contacts can reduce the number of infections, serious illness, and deaths.

Coronaviruses (CoV) were first identified 1960s. The virus was named Corona due to the specific appearance of crown like sugary proteins that surround the particle. The CoV has the longest genome of any RNA-based viruses. CoV are commonly found in animals and it is possible to transmit some of the viruses to humans. Bats are a natural host of CoV, but they are not the only animal with the ability to transmit the virus to humans. The Middle East Respiratory Syndrome Coronavirus (MERS-CoV) has been found to be camel to human transmission while the Severe Acute Respiratory Syndrome Coronavirus-1 (SARS-CoV-1) is civet cat to human transmission (European Centre for Disease Prevention and Control, 2020).

## Human Coronavirus Types

There are seven CoV’s which can infect humans, with four main subgroupings of CoV: alpha, beta, gamma, and delta. The seven coronaviruses able to infect humans consist of: (a) 229E (alpha coronavirus); (b) NL63 (alpha coronavirus); (c) OC43 (beta coronavirus); (d) HKU1 (beta coronavirus); (e) MERS-CoV (the beta coronavirus that causes Middle East Respiratory Syndrome, or MERS); (f) SARS-CoV (the beta coronavirus that causes Severe Acute Respiratory Syndrome, or SARS); and (g) SARS-CoV-2 (new CoV or COVID-19). The virus that causes the disease has been given a new name SARS-CoV-2 to better identify the family of the virus. The Centers for Disease Control and Prevention (CDC) uses the name COVID-19.

### Previous Strains and Transmissions

CoV infections are not unusual around the world. These infections are commonly associated with human coronaviruses 229E, NL63, OC43, and HKU1. CoV has the potential to mutate in animals and become transmittable to humans. When this occurs, it creates a new human coronavirus. This is the case for the COVID-19, SARS-CoV, and MERS-CoV (CDC, 2020).

### First Report of COVID-19

The novel Coronavirus 2019 (COVID-19) was first recognized and reported in Wuhan, Hubei Province, China on December 31, 2019 (World Health Organization [WHO], 2020). On January 30, 2020, the WHO announced COVID-19 as the sixth public health emergency requiring worldwide attention (WHO, 2020). This announcement follows the criteria used for H1N1 (2009), Polio (2014), Ebola in West Africa (2014), Zika (2016), and Ebola in the Democratic Republic of Congo (2019). Finally, on March 11th, 2020 the WHO designated the outbreak a pandemic.

### How Is It Different?

Ren et al. (2020) examined clinical data and bronchoalveolar specimens from five patients experiencing severe pneumonia related to COVID-19 admitted to the hospital between December 18 and December 29, 2020 in Wuhan, Hubei providence China. Chest radiography showed diffuse opacities and consolidation in all patients. One patient death was recorded. A new, unidentified beta CoV strain was observed in the five patients. The strain isolated was 79% nucleotide identifiable with the SARS and 51% identifiable with MERS. The COVID-19 is most closely related to a SARS like CoV found in bats.

### Transmission of Current COVID-19 Strain

COVID-19 is spread by human-to-human transmission via one on one contact or respiratory droplets. The median incubation time appears to be 4 to 5 days, but the ability to transmit the disease may extend to 14 days.

### Symptoms

Symptoms range from mild to severe and consist of cough, shortness of breath, and fever. Patients who screened positive for pneumonia associated with COVID-19 experienced high fever and persistent coughing (Huang et al., 2020). The symptoms closely resemble common symptoms of the influenza virus and clinicians can easily assume symptoms to be influenza. For more serious cases, CT (computed tomography) scans of the chest commonly demonstrate bilateral, peripheral, ill-defined, and ground-glass opacities (Shi et al., 2020). One U.S. case of COVID-19 pneumonia appeared to respond positively to remdesivir. China has developed clinical trials studying the effect of remdesivir on COVID-19 patients (Huang et al., 2020).

## How COVID-19 Spreads

COVID-19 is transmitted via person-to-person contact mainly with people who are within 6 feet of each other. They do not have to have direct contact as respiratory droplets present after an infected individual coughs or sneezes maybe inhaled into the lungs of others thus creating a possible infection. For the COVID-19 infection people are at greatest risk of transmitting the disease while they are experiencing symptoms with an incubation period of 2 to 14 days. The median incubation period in two studies of confirmed symptomatic patients was 4 and 5.1 days, respectively (Guan et al., 2020; Lauer et al., 2020). People appear most contagious at the time their symptom severity is highest, but several key characteristics of transmissibility, such as if transmission can occur prior to symptom onset, are currently unknown (Hellewell, 2020). It is possible to contract the live virus from touching a fomite (contaminated surface or object) and then transferring the virus to the mouth, nose, and so on, but the risk for this seems low (CDC, 2020). It remains unclear how easily the virus is transmitted from person to person (preliminary estimates suggest an infection rate of approximately 2.2 people), but it appears that older adults and those with underlying health conditions are at higher risk for mortality (Wu & McGoogan, 2020). Community spread has been documented in a number of countries including the United States.

### Super-Spreaders

Super-spreaders are individuals who infect disproportionately more susceptible contacts. Most individuals infected with COVID-19 may infect few or no people (Stein, 2011). The individual may or may know they are positive for the infection. Some may spread the disease by refusal for screening with the belief they are not infected or from fear of a positive screening result. Transmission can occur through travel, public outings, and family contacts before knowing the infection status. Super-spreaders may increase exposure to and burden of disease. The recent MERS-CoV outbreak in Korea in 2015 demonstrated how the effects of super-spreaders can affect the rates of transmission and create multiple secondary cases (Kim et al., 2018). This appears to be an issue with COVID-19. The CDC in Korea have attributed a sudden jump in COVID-19 cases to “Patient 31” who participated in a public gathering (Shin & Cha, 2020). Scientists have urged their country to prepare for super-spreader potential (Ebrahim & Memish, 2020).

## Prevention

Primary prevention is the best method against COVID-19. Avoiding geographical areas affected and known individuals positive for the virus is best practice. As with any pandemic, complete avoidance of exposure may not be possible. Patient education is crucial for any infectious disease, but especially when media is playing a significant role in sharing information. The CDC reports it is important to assess the actions taken related to the SARS epidemic in 2003. Countries were successful at aggressive screening, early identification, patient isolation, contact tracing, quarantine, and infection control methods. These lessons learned have been successfully applied in many countries to address COVID-19 transmissions.

### Personal

Patients should be properly educated and understand personal risk and personal methods to prevent infection. If diagnosed with COVID-19, patients should understand the need for self-quarantine or social distancing (measures to restrict when and where people can gather to stop or slow the spread of infectious disease) and other procedures to prevent transmission to others.

### Health Care Workers

Important lessons can be learned from previous outbreaks. During the outbreak of Ebola in West Africa in 2014-2016, the overall mortality unrelated to the virus increased because of a saturated health care system and death of health care workers (Elston et al., 2016), underscoring the importance of enhanced support for health care infrastructures and effective procedures for protecting health care workers from infection (Anderson et al., 2020).

During infectious disease outbreaks, organizational support has been deemed a key factor to protect the general and mental health for health care workers, and supervisors should be proactive in ensuring that staff members are supportive of colleagues who are quarantined (Brooks et al., 2020). This is especially important because quarantine can have lasting effects on health care workers. In past events after quarantine measures were lifted, many people were found to engage in avoidance behaviors. In health care workers, quarantine was associated with avoidance behaviors such as minimizing direct contact with patients and not reporting to work (Brooks et al., 2020). Furthermore, quarantine carries a greater risk of posttraumatic stress in hospital employees even 3 years later (Wu, 2009).

### Government Method

The Chinese Government’s effort to investigate and contain the outbreak has been acknowledged by the WHO as the most “ambitious, agile, and aggressive disease containment effort in history.” At the time the WHO was notified of the outbreak, only 27 cases had occurred. Within the following week, the virus was identified marking a significant improvement in the response time compared with the 2000-2003 SARS outbreak (Wu & McGoogan, 2020). The timing of the outbreak prior to China’s Lunar New Year holiday is an important cultural factor to consider. Several billion planned person-trips by planes, trains, and buses, would mean close contacts over long time intervals and across long distances after the holiday, which caused the government to act swiftly. Authorities focused on traditional public health outbreak response tactics including isolation, quarantine, social distancing, and community containment. All Lunar New Year celebrations were cancelled, and the city of Wuhan was isolated, this isolation was later extended to the whole Hubei providence. The Chinese Spring Festival holiday period was extended, and school openings were postponed. To assist in the fight against disease, more than 8,000 physicians and nurses from other provinces and cities were deployed to assist with management of this crisis. The Chinese Government has initiated at least 13 research programs as an emergency measure to study COVID-19 (Wang & Zhang, 2020).

The outbreak in Italy shocked European political leaders, who initially were slower to implement public health measures (*The Lancet*, 2020). The Emergency Medical Services of metropolitan Milan instituted a COVID-19 response team with the charge of tracking the outbreak without burdening ordinary emergency medical service activities. The team was tasked with handling of patient flow to local hospitals, transfer of patients to specialized facilities and management of emergency room overcrowding, and designed a procedural algorithm for the detection of suspected cases (Spina et al., 2020). Approximately 2 weeks after the virus first appeared, Italy became the first European country to announce nationwide travel limits, affecting approximately 60 million people, in an effort to halt the spread of the coronavirus outbreak. This measure follows the cancellation of the last 2 days of the famed Venice Carnival, nationwide closing of schools and universities, cancelling of sports events and outdoor gatherings, and a 6 p.m. curfew on bars. COVID-19 presents different challenges to low-income, middle-income, and high-income countries. Large outbreaks could easily overwhelm health systems in low- and middle-income countries, and many countries in sub-Saharan Africa, Latin America, and the Middle East are thought to be ill prepared for this pandemic (*The Lancet*, 2020).

### Travel Restrictions: Impact on Health

COVID-19 has affected travel for populations across the globe. Travel allows for greater transmission of disease as seen with COVID-19, but the inability to travel affects access to health care, access to employment, and ability to connect socially. Frequent air travelers disproportionately travel more than other ground or the occasional air travelers. The public health effect is seen as these travelers socially interact with other frequent travelers in common spaces such as hotels and airports. These patterns can increase the incidence and prevalence of infectious diseases, including high-contagion respiratory illnesses.

Hollingsworth et al. (2007) simulated cases from SARS-like and influenza-like epidemics in a population for which a small number air travel more frequently than others who also travel by air. The results showed that the frequent travelers increased the international transmission of epidemics only if they were infected before the illness became an epidemic and if the outbreak did not spread quickly.

With regard to public health, population health, and modes of transportation, it is important to understand all potential exposures and risk for exposures. Popular methods of travel include airway, waterway, railway, and roadway (U.S. Department of Transportation, 2018). Roadway accounts for the highest number of travelers, both short and long distance and has multiple venues, such as vehicle, bus, and taxi. Millions of people travel in communal settings such as buses and trains where the risk of being exposed to COVID-19 is present. However, decreasing the risk of COVID-19 to these groups has not been addressed at the same level as air travel. Airway is the most popular mode of travel for distances greater than 1,000 miles and international travel for both the business traveler and for personal pleasure. Airway travel also affects the greatest number of travelers and has been an effective, yet detrimental method of disease transmission from country to country.

As a method of prevention many countries are restricting entry or denying access to persons traveling from affected areas. Although this is an effective method of controlling an outbreak, it can also negatively affect those seeking care or traveling to care for a sick family member and places a burden on individuals of low-income countries who travel to higher income countries for work, as commonly seen in Asia and Europe. Qatar has restricted entry from 18 nations in varying ways from connections traveling through to mandating quarantine once an individual arrives. Italy recently announced a “lockdown” on travel, with all passengers entering and departing the country by air to be checked for travel exemption status. These measures have a rippling effect within the regions from facility closures countrywide to prison uprisings (Qatar Airways, 2020). According to Di Donato et al. (2020), the Italian government is preparing measures to support workers and firms across the country, with the hope of preventing “lasting damage to the supply side of the Italian economy and permanent employment losses” (p. 7).

President Trump issued a wide sweeping travel ban on March 12, 2020. The restrictions apply only to people who are not U.S. citizens who in the past 2 weeks have been in European nations that are part of the Schengen Area—the 26 countries that generally allow free and open movement across their borders: Austria, Belgium, Czech Republic, Denmark, Estonia, Finland, France, Germany, Greece, Hungary, Iceland, Italy, Latvia, Liechtenstein, Lithuania, Luxembourg, Malta, The Netherlands, Norway, Poland, Portugal, Slovakia, Slovenia, Spain, Sweden, and Switzerland (Specia, 2020). The travel ban was issued to protect public health due to the widespread travel between these noted European countries and parts of China still continuing to show high growth rates of COVID-19.

### Economic Impact

In 2007, the WHO released *WHO interim protocol: Rapid operations to contain the initial emergence of pandemic influenza*. Because of the impact of global travel, trade (to include health care) restrictions are not recommended by WHO once the global spread of disease is present (Mateus et al., 2014). Countries affected by the H1N1 influenza virus in 2009 instituted travel restrictions, but the approach was questioned and not considered effective (Bajardi et al., 2011).

In 2018, airlines flew more than 4.3 billion passengers. This equates to more than 11 million people daily. Industry trade groups are estimating an impact between $63 billion and $113 billion in worldwide airline revenue this year. The prospect of cancelled flights, lost sales, and service reductions is an example of how the economy is and will be negatively affected (Gelles & Chokshi, 2020). The economic impact due in large part to the worldwide scare of COVID-19 will affect marginalized groups disproportionately and be a barrier to health care for many people

## Public Response (Media Vs. Reality)

Public response is closely correlated to the amount of media coverage present for any event. When a health event or crisis is reported on TV, radio, or social media, misinformation can arise and lead to panic, anxiety, and mental health issues. The 2016 Zika event is an example of how media can affect health information and behaviors among the public. Chan (2018) studied the changes in the perceptions of community risk and how the population viewed protective behaviors. Researchers found that social media affected attitudes of risk perception while legacy media (TV, radio) affected perceptions of protective behaviors. The less educated population appeared to experience greater impact from the media in attempting to understand health information. Health care providers should consider the potential impact of media coverage on patient behaviors, health education, and care seeking when a health risk is present.

## Cultural Issues Related to Prevention

Culture may play a role in exposure to, early screening, and treatment of COVID-19. Cultural methods of greeting such as shaking of hands or a kiss on the face are widely adopted greetings internationally but may contribute to the spread of viruses and bacteria. A number of countries have recommended against hand shaking and other traditional forms of greeting such as kissing on the check and the “nose to nose” greeting. Encouraging patients to alter or adjust customary cultural practices as a form of primary prevention can be difficult, but a necessary tool to slow or alter the transmission of disease.

Several religious organizations across the globe are changing their practices to aid in primary prevention. Christian groups who shared a chalice or cups during Holy Communion have changed to individual cups, while others have chosen to limit or cancel worship meetings. Religious holidays such as the Jewish Purim have been postponed. Individuals are encouraged to attend smaller group gatherings, and religious leaders are suggesting worshippers participate in smaller groups or through online platforms. Specific mosques around the world are adjusting to the threat of outbreaks. Tajikistan has indefinitely stopped Friday prayers and The United Arab Emirates’ Sharia Council issued a fatwa, or a ruling on a point of law by a recognized authority, preventing those who are sick from attending prayers and services (Kaur, 2020). Cultural activities may pose a greater risk for exposure as individuals may not readily avoid such activities. Providers should be aware of those groups and be prepared to screen, treat, and follow-up with infected individuals.

### Cultural Perspectives

The complexity and impact of culture on health is widely known and accepted. When considering new diseases, epidemics, and pandemics, we must consider culture perceptions and ways they may affect how symptoms are recognized, access to care, treatment provided, and fear of stigmatization. Cross-cultural studies support that each specific culture has its own beliefs related to particular explanations for health and sickness (Kahissay et al., 2017; Workneh et al., 2018). Public health interventions should assess cultural beliefs and assumptions (Napier et al., 2014). These interventions should be addressed at the local level to encourage education and participation and ensure the interventions are culturally appropriate for the community (Shaikh & Hatcher, 2005). It is important to assess the role of culture and avoid correlating disease with questionable cultural causations. This may lead to blaming specific populations for their high prevalence rate or stigmatizing of certain groups (Sovran, 2013).

## Stigmatization

Infectious disease epidemics have been associated with stigmatization of specific ethnic groups. For example, the 1892 outbreaks of typhus and cholera were traced to Russian Jewish immigrants from Eastern Europe, resulting in stigmatization and discrimination of this group. Similarly, the Chinatown community was stigmatized due to an outbreak of the bubonic plaque, which was attributed to rats transported on a ship from Hong Kong, and the 1993 outbreak of Hantavirus in the United States was initially referred to as a Navajo disease, leading to discrimination and stigmatization of Native Americans in the region (Person et al., 2004).

Stigmatization is real and can negatively affect populations of people in seeking and accessing care and also in general public response. Patients who believe or perceive they are stigmatized against may delay seeking care, others become afraid of those believed to be sick, entire populations may be prejudiced against, and in some cases, stigmatization has led to violence against individuals and groups. Public health interventions should mitigate stigma while caring for individuals, families, and communities (Perry & Donini-Lenhoff, 2010). Addressing stigma and discrimination targeted toward individuals affected by COVID-19 and groups at higher risks is a priority for public health and health care providers.

Because of stigmatization and the fear of being labeled as someone who carries an infectious disease many at risk populations may not seek care until symptoms are unmanageable or may not seek care at all. During the 2003 SARS outbreak, worldwide discrimination against Asian populations was prevalent (Person et al., 2004) and affected care seeking behaviors and mental health of many people of Asian descent.

Researchers in Asia studied the effects of stigma associated with HIV/AIDS, TB, and SARS. They determined,

More efforts should be placed in strategically changing the attributions made by the public towards infectious diseases. In so doing the public would develop more acceptable attitudes towards the diseases and the affected individuals . . . For preventive programs of infectious diseases to be effective, their associated stigma must be actively addressed. (Mak et al., 2006, p. 1921)

Public health efforts to mitigate, provide surveillance, and educate the public should also meet the needs of specifically affected populations (Person et al., 2004). Researchers were careful in naming this virus as to prevent any stigma. “We had to find a name that did not refer to a geographical location, an animal, an individual, or a group of people, and which is also pronounceable and related to the disease,” said Tedros Adhanom Ghebreyesus, the director general of the WHO (WHO, 2020). Scientists and public health officials must assess the response to the names of diseases as well as using words such as epidemic. Words can create stigma against geographic regions and specific populations and result biases and panic. In 2015, the WHO established criteria for naming diseases. “This may seem like a trivial issue to some, but disease names really do matter to the people who are directly affected,” said Keiji Fukuda.

We’ve seen certain disease names provoke a backlash against members of particular religious or ethnic communities, create unjustified barriers to travel, commerce and trade, and trigger needless slaughtering of food animals. This can have serious consequences for people’s lives and livelihoods. (WHO, 2015)

The permanent name of any novel human disease is determined by the International Classification of Diseases and is managed by the WHO.

Stigma has been a major theme throughout the literature on infectious disease outbreaks and specifically surrounding quarantine measures. Quarantined individuals are more likely to report stigmatization and social rejection including avoidance, withdrawing social invitations, and making critical comments, suggesting stigma may specifically be surrounding people who are quarantined (Brooks et al., 2020). During the Ebola epidemic in Liberia, it was found that stigma could lead to disenfranchisement of minority groups, as those under quarantine were often from different ethnic groups, religions, or tribes, and perceived as dangerous (Pellecchia et al., 2015).

Data on COVID-19 from China, South Korea, Italy, and Iran suggest that the mortality increases significantly with age and those with underlying comorbidities. Targeted social distancing for these groups could be an effective way to reduce morbidity and mortality (Anderson et al., 2020), but could inadvertently increase stigma for affected populations. As health care providers, we must be aware of the potential for stigmatization of populations affected by COVID-2019 as well as the potential psychological consequences of prolonged quarantine not only on the general population but health care workers as well. Furthermore, it is important to consider the real economic impact related to lost workdays due to quarantine and social isolation efforts as well as travel restrictions that may negatively affect access to care and ability to pay for care. Efforts geared toward general education about the disease and the rationale for quarantine and public health information provided to the general public can reduce stigmatization. Media reporting is a powerful tool to influence public opinion and has contributed to stigmatization in prior outbreaks (Brooks et al., 2020).

## Conclusion

As stated by the CDC (2020), this situation is constantly changing and we are continuing to learn more about the virus, disease pathology, and tertiary effects. The cases of COVID-19 will continue to rise and the virus will be sustainable for future infections. Timely and appropriate public health interventions along with proper screening, treatment, and follow-up for affected individuals and close contacts can reduce the number of infections, serious illness, and deaths. For successful management of the epidemic, effective screening, and treatment of COVID-19 in infected patients health care providers should focus public health efforts at culturally appropriate methods of education, prevention, treatment, and follow-up.
